# Development of Novel Mouse Model of Ulcers Induced by Implantation of Magnets

**DOI:** 10.1038/s41598-017-05250-y

**Published:** 2017-07-07

**Authors:** Yuriko Takeuchi, Koji Ueno, Takahiro Mizoguchi, Makoto Samura, Takasuke Harada, Atsunori Oga, Tomoaki Murata, Tohru Hosoyama, Noriyasu Morikage, Kimikazu Hamano

**Affiliations:** 1 Department of Surgery and Clinical Sciences, Yamaguchi University Graduate School of Medicine, Ube, Japan; 2 Center for Regenerative Medicine, Yamaguchi University Graduate School of Medicine, Ube, Japan; 3 Department of Molecular Pathology, Yamaguchi University Graduate School of Medicine, Ube, Japan; 4 Institute of Laboratory Animals, Yamaguchi University, Ube, Japan

## Abstract

We developed a novel mouse model of human refractory cutaneous ulcers that more faithfully reflects pathology and evaluated the effects of mixed cell sheets comprising peripheral blood mononuclear cells and fibroblasts, which we previously developed for treating refractory cutaneous ulcers. Model development involved sandwiching the skin between two magnets, one of which was implanted under the skin for 7 consecutive days. This magnet-implanted ulcer model produced persistently large amounts of exudate and induced the infiltration of the ulcer with inflammatory cells. The model mice had a thicker epidermis and impaired transforming growth factor-β (TGF-β) signaling followed by SMAD2 down-regulation, which causes epidermal hyperplasia in chronic ulcers. Impaired TGF-β signaling also occurred in the ulcers of critical limb ischemia patients. Mixed cell implantation in this ulcer model reduced TNF-α and IL-6 levels in the tissues surrounding the mixed cell sheet-treated ulcers compared with controls or mice treated with trafermin (FGF2). Seven days after commencing therapy, the epidermis was thinner in mice treated with the mixed cell sheets than in controls. This model may therefore serve as a clinically relevant model of human ulcers, and our mixed cell sheets may effectively relieve chronic inflammation and inhibit refractoriness mechanisms.

## Introduction

Many patients worldwide suffer from refractory skin ulcers caused by various pathologies^1–3^, including congestion such as that caused by venous insufficiency and ischemic disorders like arteriosclerosis obliterans (ASO), Buerger’s disease, blue toe syndrome, diabetes mellitus, and decubitus. It is known that in chronic refractory skin ulcers, there is impaired secretion of cytokines and growth factors, recruitment of stem cells^4^, and inadequate macrophage switching^5^. Deregulation of epidermal activation and differentiation, including attenuation of epidermal growth factor (EGF) and transforming growth factor-β (TGF-β) receptor signaling^6–8^, also reportedly causes refractory mechanisms, particularly in venous ulcers, there are few papers which show the molecular pathophysiology of ischemic ulcer.

Wound healing is a highly regulated, complex process requiring an appropriate influx of inflammatory cells, granulation tissue that forms a network of capillaries induced by angiogenesis, fibroblast migration, fibroblast conversion to myofibroblasts, collagen deposition, and re-epithelialization. Effective cytokine or growth factor concentrations are essential for wound healing^2^. Unlike acute wound healing in healthy individuals, refractory cutaneous ulcers occur from various causes and therefore are more complex. Despite numerous investigations on pathology of the refractory chronic ulcers, the mechanism remains unclear. Therefore, it is difficult to develop suitable animal models of refractory skin ulcers, limiting new therapy development.

Animal models of ulcers^2, 9^ are developed as follows: simple excision, cutaneous ischemia and reperfusion injury caused by intermittent skin sandwiching between magnets^10, 11^, ischemia induced by a skin flap or supplemental vessel ligation^12, 13^, diabetic mice^14^, and infected wounds^15^. However, because of the unique panniculus carnosus layer of mouse skin, which promotes rapid wound contraction^9^, no model exhibits a dramatic delay in wound closure that reflects the clinical manifestation. Although skin contraction can be minimized to some extent by splinting a silicone ring around the ulcer^16^, these characteristics represent important differences compared with human wound healing via re-epithelialization and granulation tissue formation. Furthermore, because dry wounds contract more rapidly with high closure rates^17^, detachment of their dressings from the wound due to their aggressive movement makes it difficult to precisely evaluate the healing process. Then, we focused on the molecular environment in the wound to reproduce an actual human refractory skin ulcer than on the wound closure speed to develop a mouse model of cutaneous ulcers.

## Results

### Macroscopic and histological findings

**Figure 1:**
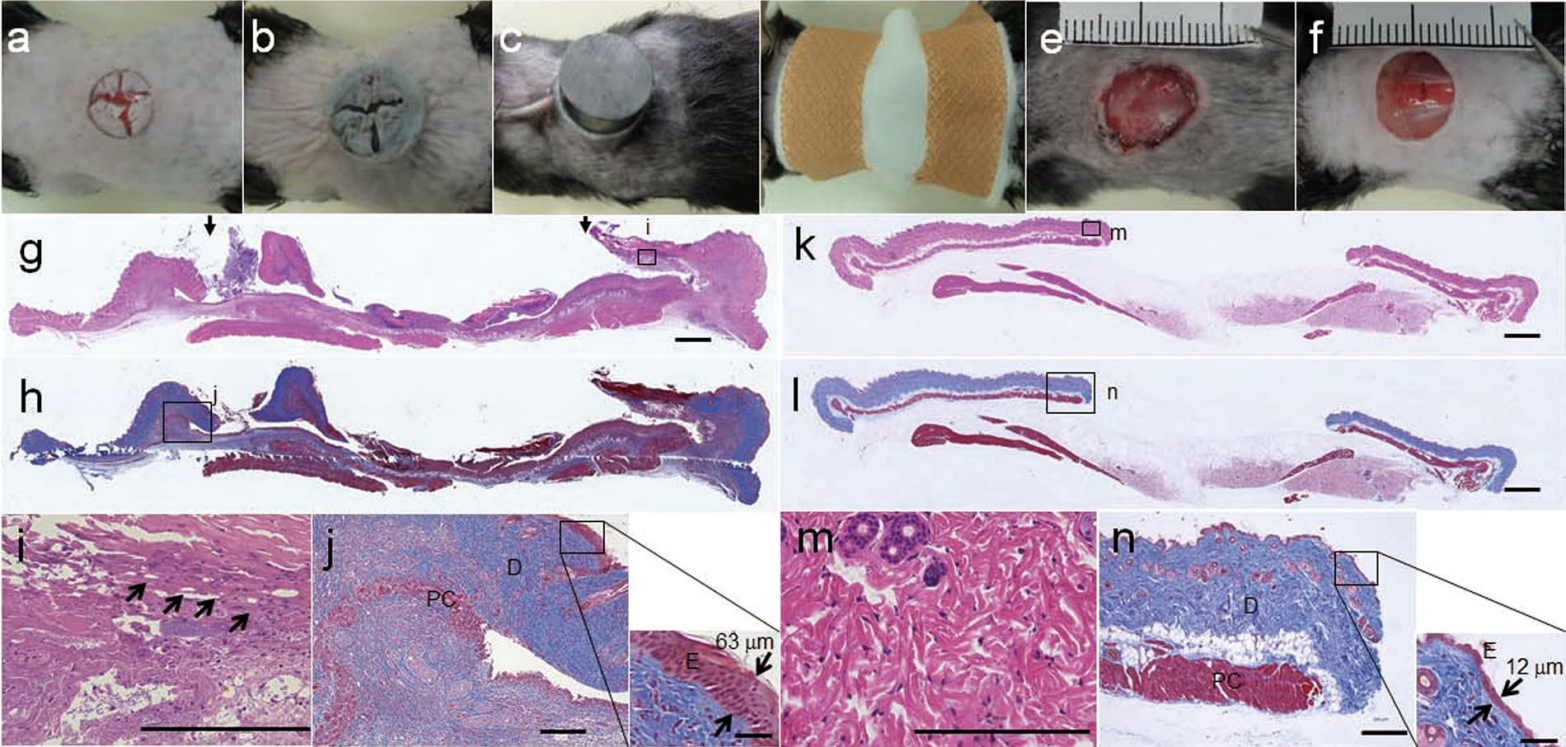
Magnet-implanted ulcer model. (**a**–**e**) Methods used to prepare magnet-implanted ulcer and (**f**) simple skin defect models. (**g**,**h**) Representative histologies of ulcers 7 days after magnet implantation. (**g**) Hematoxylin & Eosin (H&E) staining, scale bar = 1000 µm, and (**h**) Masson trichrome staining, scale bar = 1000 µm. Two arrows in (**g**) show the edges of ulcer. (**i**) The enlarged image enclosed in a square in (**g**) indicated by the letter “i,” scale bar = 200 µm. The black arrows show coagulation necroses. (**j**) The magnified image of the area enclosed in the square in (**h**) indicated by the letter “j,” scale bar = 200 µm. (E, epidermis; D, dermis; PC, panniculus carnosus layer). (**k**) (**l**) Representative histology of the simple skin defect. (**k**) Hematoxylin & Eosin (H&E) staining, scale bar = 1000 µm and (**l**) Masson trichrom staining, scale bar = 1000 µm. (**m**) The enlarged image enclosed in the square in (**k**) indicated by the letter “m,” scale bar = 200 µm. (**n**) The image enclosed in the square in (**l**) indicated by the letter “n,” scale bar = 200 µm. (E: epidermis; D, dermis; PC, panniculus carnosus layer.

A model of magnet-implanted ulcers was developed as follows: A cross section was made on the back of the mouse (Fig. 1a), and one ferrite magnet (f10 mm, 5-mm thick) was implanted under the skin through the incision (Fig. 1b). Next, the incised skin was sandwiched between the other magnet (Fig. 1c). The torso was then wrapped with cotton, and the magnets were secured with a surgical tape before forming the ulcers (Fig. 1d). After 7 days, the two magnets were removed, the sandwiched skin dislodged spontaneously, generating an ulcer (Fig. 1e). Compared with the model of a simple skin defect, which was prepared by excising the skin with a surgical scissor (Fig. 1f), the impaired edge and contaminated bottom of ulcers were grossly observed.

Skin tissues were harvested from 13 magnet-implanted models and processed for histological analysis after the ulcers formed. Hematoxylin and eosin (H&E) and Masson trichrome (MT) staining revealed that the panniculus carnosus and shallower layers were defected after removing the magnets and skin 7 days after placing the magnets (Fig. 1g–i). The epidermis and dermis were thicker at the ulcers’ edges (Fig. 1j) than at the simple skin defect’s edge (Fig. 1n). Thickness of epidermis was 63 μm in the simple defect and 12 μm in the magnet ulcer. Coagulation necroses were observed in the dermal layer, which were likely caused by the ischemic injury (Fig. 1g and i) and absent in simple defect (Fig. 1k and m).

### Ulcer exudates produced by magnet-implanted ulcers

**Figure 2:**
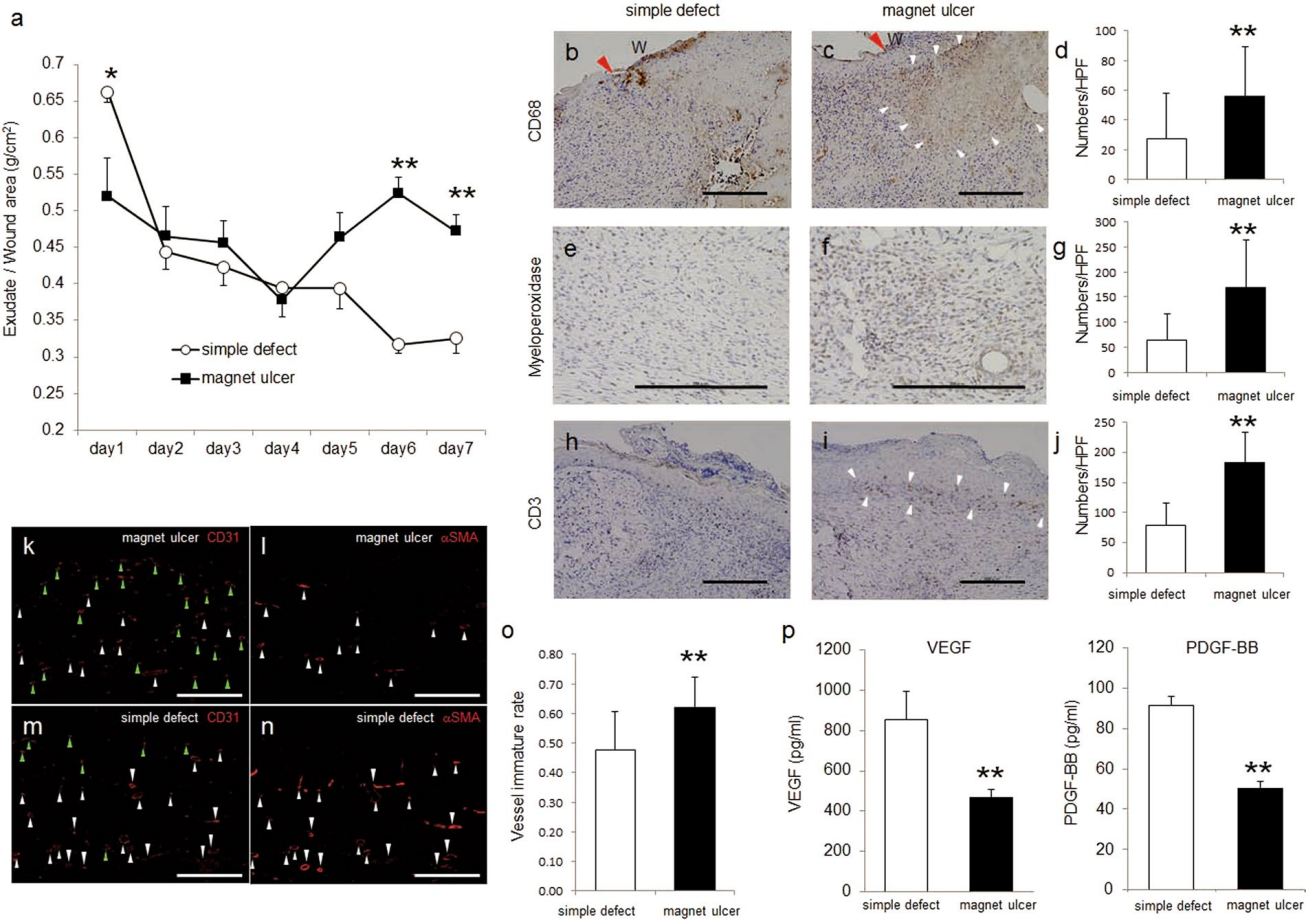
Inflammatory properties of the magnet-implanted ulcer model. (**a**) Exudate from wounds was analyzed daily by measuring the weights of dressings and correcting the ulcer areas 7 days after forming ulcers using each model. *P < 0.05 versus simple defect, **P < 0.01 versus simple defect. (**b**,**c**,**e**,**f**,**h**, and **i**) Immunohistochemical analysis of macrophages (CD68), neutrophils (myeloperoxidase), and T lymphocytes (CD3) on day 7 (scale bar = 200 µm). In (**b**) and (**c**), red arrowheads indicate the wound edge. In (**c**), white arrowheads indicate the infiltration area macrophages. (W, wound) In (**i**), white arrowheads indicate the infiltration area of T lymphocytes at the bottom of the epidermis. (**d**,**g**, and **j**) Eight of high-power fields in three serial sections were counted and averaged for macrophages, neutrophils, and T lymphocytes respectively. (**k**–**n**) Immunohistochemical analysis of CD31 and α-SMA in the magnet-implanted ulcer model and the simple skin defect. (**k**,**l**,**m** and **n)** were the serial sections. The white arrowhead indicates CD31^+^ α-SMA^+^ vessels and the green arrowhead indicates CD31^+^ α-SMA^−^ vessels (scale bar = 200 µm). (**o**) comparison of the CD31^+^ α-SMA^−^/all CD31^+^ ratio as the vessel immature rate between magnet ulcers and simple defects (**p**) ELISA analysis of VEGF and PDGF-BB levels in supernatants of homogenates of the skin around the wounds. Values are expressed as mean ± SD. **P < 0.01 versus simple defect.

An excessive exudate represents an important factor contributing to refractory ulcer development^18^. Therefore, exudate weights were determined each day. Exudate weights from magnet-implanted ulcers increased after day 5 and were greater than those of simple skin defects on days 6 and 7, although the amount of the former was lower on day 1 (Fig. 2a).

### Inflammatory cell infiltration at the edges of magnet-implanted ulcers

An excessive exudate correlates with vascular permeability, which is followed by inflammatory cell infiltration into the ulcer. An excessive exudate is a characteristic of models of magnet-implanted ulcers. Therefore, to better understand the underlying mechanism, we performed immunohistochemical analyses of ulcer tissues on day 7 and found that inflammatory cells comprised numerous macrophages, neutrophils, and T lymphocytes. Macrophages were mainly observed at the ulcer bottom (Fig. 2b and c), neutrophils were observed throughout (Fig. 2e and f), and T lymphocytes mainly localized to the epidermal basal layer at the ulcer edge (Fig. 2h and i). There were more inflammatory cells in a magnet ulcer than in a simple defect (Fig. 2d, g, and j). On day 7, we investigated the presence of immature vessels, which are associated with vascular permeability, and not lined by vascular smooth muscle. For this, we performed immunohistochemical analyses of CD31 and α-SMA expression, which serve as markers of vascular endothelial and vascular smooth muscle cells, respectively. There were more CD31-positive and α-SMA-negative immature vessels in the magnet ulcer (Fig. 2k and l) than in the simple defect (Fig. 2m and n). The vessel immature rates were calculated by dividing the numbers of CD31-positive and α-SMA-negative vessels by the numbers of all CD31-positive vessels; these rates were compared between the simple defect and magnet ulcer (Fig. 2o). On day 7, vascular endothelial growth factor (VEGF) and PDGF-BB expression levels were lower in magnet-implanted ulcers than in simple skin defects (Fig. 2p).

### Epidermal thickness on the edge of magnet-implanted ulcers

**Figure 3:**
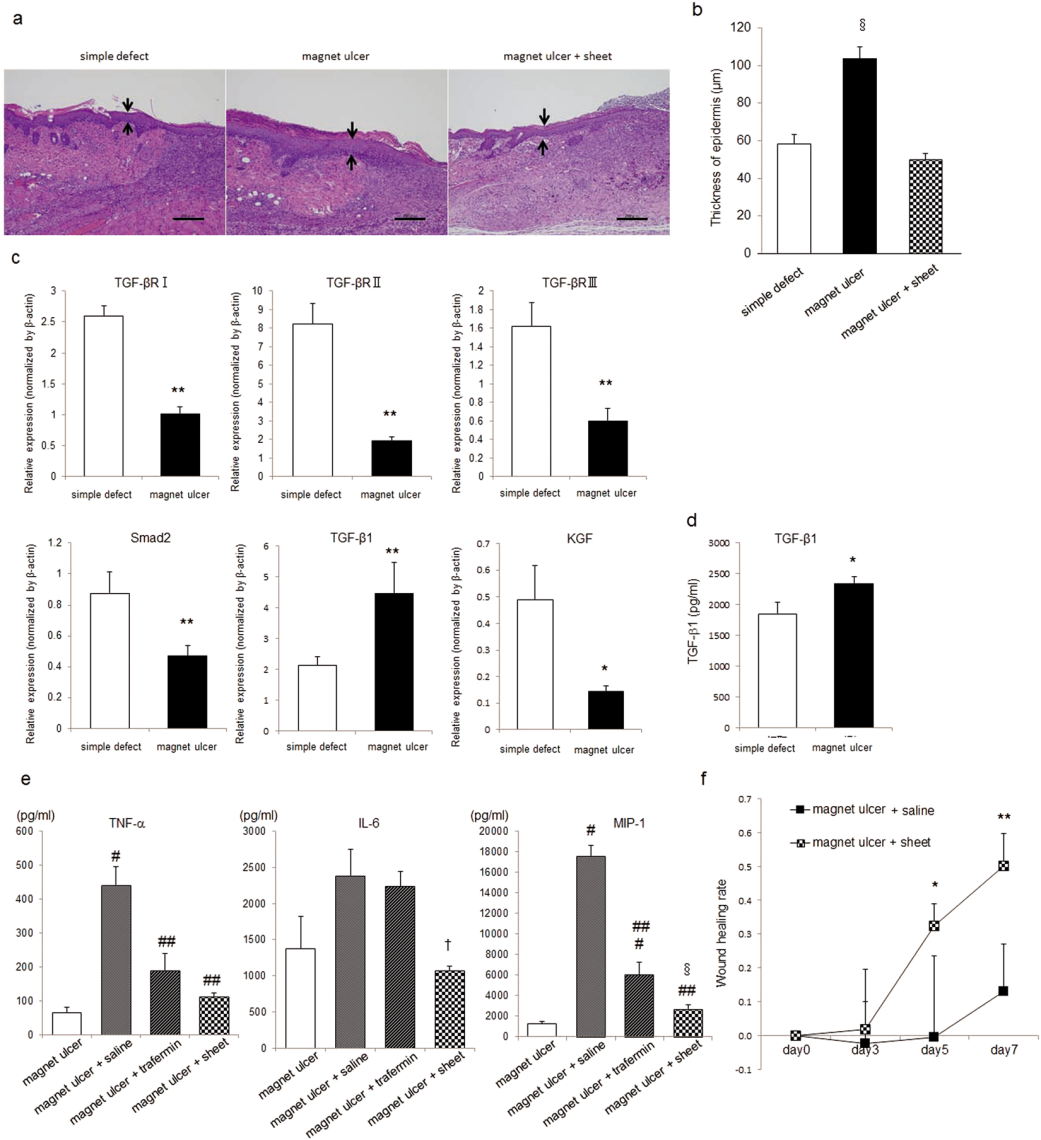
Analysis of the epidermis in the magnet-implanted ulcer model and the effect of mixed cell sheets. Skin around the wounds collected 7 days after ulcer formation was subjected to histological analysis to evaluate epidermal thickness around the wounds, and qPCR and ELISA were used to determine associated factor expression. (**a**) In each representative tissue section of a simple defect, magnet-induced ulcer, and magnet-induced ulcer plus the mixed cell sheet, the sets of two arrows show the epidermal thickness (scale bar = 200 µm). (**b**) Epidermal thickness was determined using Image J. Two high-power fields in three serial sections were measured and averaged. Values are expressed as mean ± SD. §P < 0.01 vs simple defect and mixed cell sheet on the magnet-induced ulcer. (**c**) Levels of mRNAs encoding TGF-βRI, TGF-βRII, TGF-βRIII, SMAD2, TGF-β1, and KGF were determined using qPCR using ACTB as the endogenous control. Values are expressed as mean ± SD. *P < 0.05 vs simple defect, **P < 0.01 vs simple defect. (**d**) ELISA was used to determine TGF-β1 levels in the supernatants of homogenates of the skin around wounds. Values are expressed as mean ± SD. **P < 0.01 versus simple defect. (**e**) ELISAs of TNF-α, IL-6 and MIP-1 levels in supernatants of homogenates of the skin around wounds. Magnet-induced ulcer means the skin collected 7 days after magnet implantation as control (day 0). Magnet-induced ulcer plus saline/trafemin/sheet showing the skin treated on days 0 and collected on day 1. Values are expressed as mean ± SD. ^#^P < 0.01 vs magnet-induced ulcer, ^##^P < 0.01 vs magnet-induced ulcer plus saline, ^†^P < 0.05 versus magnet-induced ulcer plus saline. (**f**) Comparison of wound healing rate between the magnet -induced ulcer plus saline and sheet. *P < 0.05, **P < 0.01 versus magnet ulcer + saline.

The impaired edge of magnet-implanted ulcers was enlarged and the epidermis was hyperplastic compared with simple skin defects on day 7. The epidermis at the edge of magnet-implanted ulcers was thicker than that at simple skin defects (Fig. 3a and b). The levels of mRNAs encoding TGF-βRI, TGF-βRII, TGF-βRIII, and SMAD2, TGF-β signaling pathway components, were significantly lower in the skin around magnet-implanted ulcers than in the skin around simple defects (Fig. 3c). TGF-β1 mRNA and protein levels were higher in the magnet-implanted model than in the simple skin defect model (Fig. 3c and d). The levels of mRNA encoding KGF (FGF7) were significantly lower in the skin around magnet-implanted ulcers than in the skin around simple defects (Fig. 3c).

### The effect of trafermin (FGF2) and a mixed cell sheet on magnet-implanted ulcers

We applied our novel mixed cell sheets comprising PBMNCs and fibroblasts^19^ as a material for treating magnet-implanted ulcers. TNF-α, IL-6 and MIP-1 expression in magnet-implanted ulcers was elevated on the day after forming the ulcer (day 0) as a control (Fig. 3e; P < 0.01). Treatment was administered on day 0, and ulcers were collected on day 1. The application of trafermin or mixed cell sheets reduced TNF-α and MIP-1 expression compared with saline (Fig. 3e; P < 0.01). Similarly, IL-6 expression was significantly lower only in the skin around cell sheet-treated ulcers than in controls (Fig. 3e; P < 0.05). The effect of mixed cell sheets on the epidermis around ulcers was apparent on day 7, when the epidermis was thinner in cell sheet-treated ulcers than in untreated ulcers (Fig. 3a and b; P < 0.01). When comparing the wound healing rate, which was calculated by dividing the decreased area from day 0 by the area on day 0, between the magnet-implanted ulcers treated with the mixed cell sheets and the saline (control), the former provided significantly higher healing rate (Fig. 3f; day5; P < 0.05, day7; P < 0.01).

### Expression patterns of mRNAs encoding signaling molecules in ischemia-induced human ulcers and fibroblasts

**Figure 4:**
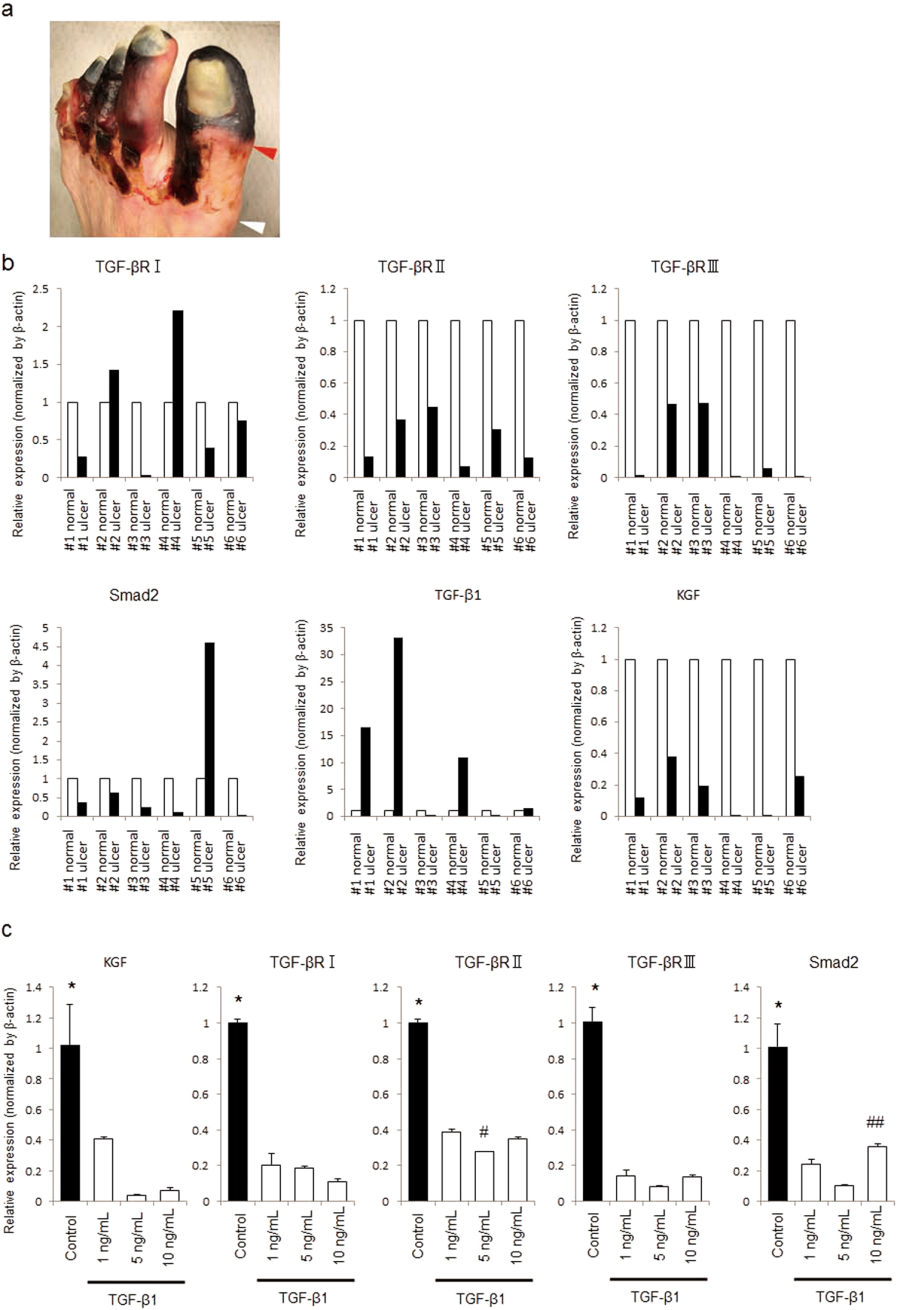
Characteristics of human ulcers caused by ischemia. (**a**) Images of the ischemic ulcer of patient 3. After amputation, the skin around the ulcer (red arrowheads) and the amputated stump as a control (white arrowheads) was collected. (**b**) Levels of mRNAs encoding TGF-βRI, TGF-βRII, TGF-βRIII, SMAD2, TGF-β1, and KGF were analyzed using qPCR with ACTB as an endogenous control and are presented relative to those of normal skin. (**c**) Recombinant TGF-β1 reduced the levels of mRNA encoding KGF, TGF-βRI, TGF-βRII, TGF-βRIII, and SMAD2 in human fibroblasts. Values are expressed as mean ± SD. *P < 0.01 vs all samples, ^#^P < 0.01 vs 1 ng/mL and 10 ng/mL, ^##^P < 0.05 vs 5 ng/mL.

Table 1 presents the characteristics of six patients who underwent limb amputation for nonhealing ulcers caused by ASO or blue toe syndrome (Table 1). Skin tissues around the ulcer and the sharply amputated stump (control) were collected (Fig. 4a). For TGF-βR II, TGF-βR III, and KGF, skin around ulcers had a significantly lower mRNA expression than normal skin. For TGF-βR I, SMAD2, and TGFβ-1, 4 of 6 patients showed the same expression patterns; mRNA expression of TGF-βR I and SMAD2 was lower in skin around ulcers than in normal skin, while the expression of TGFβ-1 expression was higher (Fig. 4b). Treating human fibroblasts with recombinant TGF-β1 reduced the levels of mRNAs encoding KGF, TGF-βRI, TGF-βRII, TGF-βRIII, and SMAD2 (Fig. 4c).

**Table 1:** Patient characteristics.

Case	Age	Sex	Cause of ulceration	Ulcer suffering duration	Topical treatment	Dressing
#1	83	F	^1^ASO, ^2^DM	2 years	None	Gauze
#2	91	F	^1^ASO	6 months	None	Gauze
#3	86	M	Blue toe syndrome	2 months	Prostandin ointment	Gauze
#4	73	M	Blue toe syndrome, ^2^DM	1 month	None	Silicon gel dressing
#5	71	F	^1^ASO	1 month	None	Hydrogel dressing
#6	76	M	^1^ASO, ^2^DM	2 months	None	Gauze

^1^ASO: atherosclerosis obliterans, ^2^DM: diabetes mellitus.

## Discussion

We developed a novel mouse model of ulcers relevant to refractory cutaneous ulcers in humans. Magnet-implanted mice exhibited coagulation necroses in the dermal layer, reflecting ischemic injury, suggesting that continuous 7-day-compression by magnets caused vascular skin insufficiency around magnets by inhibiting blood flow across the skin. This model is similar to models of diabetes mellitus with microvascular insufficiency^20^. Magnet-implanted ulcers showed persistent and larger amounts of exudates than ulcers formed by introducing a simple skin defect, and numerous immature vessels and inflammatory cells were observed in ulcers formed by implanted magnets. Excessive exudates are associated with inflammatory responses caused by reactions to foreign bodies such as the magnets used here, and inflammation is associated with an increase in vascular permeability and cellular infiltration^21^.

Our data support the hypothesis that hyperpermeability of immature vessels and infiltration of increased inflammatory cell numbers caused excessive exudates in magnet-implanted mice. Excessive exudates from ulcers cause maceration in the skin at the ulcer edge, and ulcers become refractory to treatment^18^. In contrast, an appropriate amount of exudate mediates ulcer healing^22, 23^, raising the possibility that the excessive exudate observed here interfered with optimum wound healing. VEGF and PDGF-BB promote angiogenesis, and PDGF-BB plays an important role in newly formed vessel maturation^20^. Here VEGF and PDGF-BB levels were lower in magnet-implanted ulcers than in simple skin defects, which inhibited angiogenesis.

Evidence suggests that TGF-β signaling down-regulation contributes to epidermal hyperproliferation and refractoriness of human venous ulcers to treatment^6–8^, and venous ulcer fibroblasts exhibit decreased proliferative responses to TGF-β1^24^. We showed that epidermal hyperproliferation and TGF-βRI, TGF-βRII, TGF-βRIII, and SMAD2 expression down-regulation were detected in magnet-implanted ulcers. TGF-βRII and TGF-βRIII levels were lower in all ischemic ulcer patients, and TGF-βRI and SMAD2 expression was reduced in 4 of 6 patients. Although TGF-β1 levels are reduced in chronic venous ulcers^25, 26^, 4 of 6 patients studied here with ischemic ulcers expressed markedly increased TGF-β1 levels in the skin around the ulcer compared with the normal tissue. TGF-β1 mRNA and protein levels were higher in magnet-implanted ulcers than in simple skin defects. We showed that recombinant TGF-β1 reduced TGF-βRI, TGF-βRII, TGF-βRIII, and SMAD2 expression in human fibroblasts, suggesting that continuous higher TGF-β1 levels in the skin around the ulcer than in normal skin contribute to TGF-β signaling down-regulation in human ischemic ulcers and magnet-implanted mice. Impaired TGF-β signaling may serve as a common mechanism more frequently in refractory chronic ulcers than in venous ulcers.

KGF, expressed by fibroblasts but not primary keratinocytes^27^, stimulates keratinocyte migration and proliferation and promotes wound re-epithelialization^28^. Here KGF expression decreased in magnet-implanted ulcers and ischemic ulcer patients, and recombinant TGF-β1 reduced KGF expression in human fibroblasts. Although a functional relationship between KGF and TGF-β1 has not been identified, continuous stimulation by TGF-β1 may affect the KGF expression pattern in magnet-implanted mouse ulcers and human ischemic ulcers and KGF downregulation may lead to optimal re-epithelialization inhibition.

Our data indicate that the magnet-implanted model is similar to the general refractory ulcers characterized by excessive exudates and prolonged inflammation and showed the aberrant epidermal behavior clinically observed in the venous ulcers. Although it has already been reported that venous ulcer has deregulation of TGF-β signaling, our present human data in ischemic ulcer showed same genomic profiling in relation to the attenuation of TGF-β signaling. Our magnet ulcer model also had the same pattern of TGF-β signaling as the ischemic ulcer. Although we consider that this model may be more clinically relevant to human skin ulcers, there is a limitation, that is, magnet-implanted ulcer can be healed eventually. Therefore, this model is not perfectly representative of a clinical human refractory skin ulcer, which is extremely hard to cure; our future studies will attempt to overcome this problem. Similarly, clinical therapy using the magnets—magnetic compression anastomosis for biliary obstruction or bowel stenosis—has been reported for the patients who could not be treated with conventional therapies^29^. The mechanism is that two abdominal hollow organs, such as bowel and biliary duct or bowel and bowel, are sandwiched by two magnets; sandwiched area drops out, and then, the two organs are attached each other at the edge of the magnets. However, this reportedly takes 10 days or more, implying that healing after magnetic compression needs long time. From this phenomenon, we regard our magnet-implanted ulcer model as reproduction of human refractory cutaneous ulcers.

Finally, we investigated the effects of cell sheets comprising PBMNCs and fibroblasts, which we developed as a technique for treating refractory cutaneous magnet-implanted ulcers^19^. Inflammatory cytokine TNF-α, IL-6 and MIP-1 levels were lower in ulcers treated with mixed cell sheets than in untreated ulcers. Although evidence indicates that mixed cell sheets dramatically promote wound healing just after the inflammatory phase, together with the present and previous data^19^, we suggest that mixed cell sheets inhibit inflammatory cytokine activities during inflammation and induce efficient wound healing. Moreover, Mixed cell sheets reduced epidermal thickness on day 7 and provided the significant higher healing rate of magnet-implanted ulcer on day 5 and day 7. Together, these data indicate that mixed cell sheets serve as a promising agent for treating cutaneous ulcers.

## Materials and methods

### Animals

Male C57BL/6 mice were purchased from Japan SLC, Inc. (Shizuoka, Japan). The Institutional Animal Care and Use Committee of Yamaguchi University approved all animal procedures (#31-093). The animal experiments were conducted in accordance with the approved guidelines.

### Cutaneous wound model

Mice were anesthetized via 1.5% isoflurane inhalation during the surgical procedure. The surgical instruments and magnets were sterilized using dry heat and EOG gas sterilization. The mice were shaved, depilated, and disinfected with 75% ethanol.

### Exudate quantification from the wounds

Magnet-implanted ulcers and simple skin defects were covered with hydrocolloid dressing, Absocure (Nitto Medical CO, Japan), which maintained high liquid absorption and retention, and the trunks of mice were fixed with an elastic adhesive bandage (Nichiban, Japan), respectively. The dressings were exchanged and their weights were measured daily before and after exchange. The exudate weight was calculated by subtracting the weight of dressing before adding the plaster from the weight after removal, and divided by the ulcer area at that point, which was measured using ImageJ to correct the influence of differences in the wound closure rates between the two models.

### Histological analysis

To compare the features of the magnet-implanted ulcers with those of the simple skin defects, the difference in inflammatory cell infiltration, immature vessel formation, and epidermal thickness around the ulcers 7 days after the production of each ulcer were assessed. The skin around the ulcers was resected, fixed in 10% paraformaldehyde, and embedded in paraffin. Sections (3 µm) were stained with H&E and MT or processed for immunostaining. For immunohistochemical analysis of inflammatory cells, deparaffinized sections were subject to epitope retrieval using Target Retrieval Solution (DAKO Cytomation A/S, Copenhagen, Denmark) and treated with a biotin-blocking system (DAKO Cytomation A/S), an endogenous peroxidase-blocking reagent (DAKO Cytomation A/S), and a protein blocking solution (DAKO Cytomation A/S). Sections were then incubated with a mouse monoclonal antibody specific for CD68 (ab31630, Abcam), a rabbit anti-myeloperoxidase polyclonal antibody (ab9535), or a rat anti-CD3 monoclonal antibody (ab11089) for 2 h at room temperature. Sections were then incubated sequentially with a biotinylated goat anti-mouse IgG secondary antibody (ab98725), a biotinylated goat anti-rabbit IgG secondary antibody (ab98725), or a biotinylated goat anti-rat IgG secondary antibody (ab98395) for 1 h at room temperature and then with horseradish peroxidase-conjugated avidin–biotin complexes (ab74034). Sections were washed three times with phosphate-buffered saline, color was developed using 3,3′-diaminobenzidine and hydrogen peroxide, and the sections were then counterstained with hematoxylin. For immunohistochemical analysis of vessels, the sections were deparaffinized, subjected to epitope retrieval, and blocked with the protein blocking solutions described above. The antibodies used were as follows; anti- α-SMA-Cy3 antibody (C6198, Sigma-Aldrich), anti-CD31 antibody (ab28364, Abcam), and Dylight 550-conjugated goat anti-rabbit IgG secondary antibody (ab96884). To assess the epidermal thickness, HE samples were observed, and the epidermal thickness at the ulcer edge was measured using ImageJ.

### Analysis of mRNA expression levels of factors associated with epidermal changes

mRNA expression levels of factors associated with epidermal changes were determined using quantitative real time reverse transcriptase (qPCR) analysis. Total RNAs were extracted from the skin around the wound 7 days after forming the wound using an RNeasy Plus Mini Kit (Qiagen, Hilden, Germany). The extracted total RNA was reverse-transcribed into single-stranded cDNA with using a PrimeScript RT reagent Kit (Perfect Real Time) (TaKaRa Bio Inc, Kusatsu, Shiga, Japan) following the manufacturers’ instructions. Real-time PCR was performed using the first-strand cDNA with SYBR Select Master Mix (Thermo Fisher Scientific). The mouse primers used were shown in Table 2. We performed qPCR using a StepOnePlus Real-Time PCR System (Thermo Fisher Scientific). The qPCR parameters for cycling were as follows: 50 °C for 2 min, 95 °C for 2 min, and 40 amplification cycles at 95 °C for 3 s and 60 °C for 30 s. All reactions were performed in triplicate in a 10-μl reaction mixture. mRNA expression levels were determined using the ∆∆Ct method.

**Table 2:** Primer Sequences.

Name	Sequence	Human
	Mouse	
β-actin ^1^FWD	5′-gctcctcctgagcgcaag-3′	5′-gctcctcctgagcgcaag-3′
β-actin ^2^REV	5′-catctgctggaaggtggaca-3′	5′-catctgctggaaggtggaca-3′
TGF-βRI FWD	5′-atggttccgagaggcagaga-3′	5′-gctcaggtttaccattgcttgttc-3′
TGF-βRI REV	5′-ccatgtcccattgtctttgttg-3	5′-ttcctctccaaacttctccaaatc-3′
TGF-βRII FWD	5′-gcgggcgagactttcttca-3′	5′-gtgtggctgtatggagaaagaatg-3′
TGF-βRII REV	5′-ggactgctggtggtgtattcttc-3′	5′-gtcatggtaggggagcttgg-3′
TGF-βRIII FWD	5′-aaagccgccgaaggttg-3′	5′-agcagttacttcattcaccgaactc-3′
TGF-βRIII REV	5′-ggaaggtgctgtaaggattgga-3′	5′-ctatgttgcactttggagggaac-3′
SMAD2 FWD	5′-agcagtgaaaagtctggtgaaaaag-3′	5′-aacttcccagcaggaattgag-3′
SMAD2 REV	5′-tgcaattctgagtggtgatgg-3′	5′-ggtcacttgtttctccatcttcac-3′
TGF-β1 FWD	5′-ctgctcttgtgacagcaaagataac-3′	5′-cgtggaggggaaattgagg-3′
TGF-β1 REV	5′-cggttcatgtcatggatggtg-3′	5′-cggtagtgaacccgttgatg-3′
KGF FWD	5′-aaagggacccaggagatgaag-3′	5′-tgaacaaggaaggaaaactctatgc-3′
KGF REV	5′-cactttccacccctttgattg-3′	5′-gctgatgcatatgtgttgtaatgg-3′

^1^FWD: forward, ^2^REV: reverse.

### Growth factor and cytokine quantification

Analysis of growth factors contained in the skin around the magnet-implanted ulcers was performed 7 days after ulcer production. To analyze cytokines in tissues after treatment of the magnet-implanted ulcers, the skin around the ulcer treated with trafermin or mixed cell sheets was collected the following day. The skin around the flesh of the ulcers just after the 7-day implantation of the magnet and the skin around the untreated ulcers were collected as controls. Protein was extracted from the tissues using lysis buffer, and an ELISA was performed to measure VEGF, PDGF-BB, TNF-α, IL-6 and MIP-1α production (R&D Systems) according to the manufacturer’s protocol.

### Cell sheet preparation and transplantation

PBMNCs were isolated from mouse peripheral blood using Lympholyte-M (Cedarlane Laboratories Ltd., Hornsby, Ontario, Canada) and cultured in CTS AIM-V (Thermo Fisher Scientific, Waltham, MA, USA) containing 10% fetal bovine serum (Thermo Fisher Scientific). Fibroblasts were isolated from the tails of mice using collagenase (Wako, Osaka, Japan) and cultured in AIM-V (Thermo Fisher Scientific) containing 10% fetal bovine serum. For mixed cell sheets, 1 ml PBMNCs (2 × 10^6^ cell/ml) and 1 ml fibroblasts (1.25 × 10^5^ cell/ml) were added to the same well in UpCell 24 multiwell plates (CellSeed Inc., Tokyo, Japan). For hypoxic preconditioning, cells were incubated for 2 days under normoxic conditions (37 °C in 20% O_2_ and 5% CO_2_) and then for 1 day under hypoxic conditions (33 °C in 2% O_2_ and 5% CO_2_). Mixed cell sheets were aspirated using a 1000 µl pipet and transplanted onto magnet-implanted ulcers. The ulcers were covered with UrgoTul (Laboratories Surgo, France) and Derma Aid (ALCARE, Japan) and then secured with an elastic adhesive bandage (Nichiban, Japan).

### Human samples and mRNA expression analysis

We used discarded tissues that were resected during minor amputation of the toes of six refractory cutaneous ulcer patients caused by progressive ASO. Informed consent was obtained from each patient, and the Institutional Review Board of Yamaguchi University Hospital approved the research protocol (H27-193). All investigations were conducted in accordance with the Declaration of Helsinki. Total RNAs were extracted from the skin around the ulcer, and the sharply amputated stump served as the control. Total RNAs were extracted from the human fibroblast using an RNeasy Plus Mini Kit (Qiagen, Hilden, Germany). The extracted total RNA was reverse-transcribed into single-stranded cDNA using a PrimeScript RT reagent Kit (Perfect Real Time) (TaKaRa Bio Inc.) following the manufacturers’ instructions. Real-time PCR was performed using first-strand cDNA and SYBR Select Master Mix (Thermo Fisher Scientific). The human primers used were shown in Table 2.

### Human fibroblast preparation and recombinant TGF-β1 administration

Fibroblasts were isolated from the oral mucosa of healthy volunteers using collagenase (Wako, Osaka, Japan) and cultured in CTS AIM V Medium (Thermo Fisher Scientific) containing 5% serum from an individual’s blood, which was prepared using a Serum Collection Set (JMS Co., Ltd., Hiroshima, Japan). Twenty-four hours after human fibroblast (2 × 10^4^ cell/well) was seeded into 24 multiwell plates, and 1 ng/mL, 5 ng/mL, and 10 ng/mL recombinant TGF-β1 were added. After 24 h incubation, total RNAs were extracted from the human fibroblasts, and total RNAs and qPCR was performed as described above.

### Statistical analysis

Data were analyzed using Stata/SE 12.1 (StataCorp, USA). The statistical significance of differences in data between two groups was determined using two-tailed unpaired -tests. Statistical significance among multiple groups was analyzed using one-way ANOVA followed by a Bonferroni post-hoc test. P-values < 0.05 were considered statistically significant. Data are expressed as mean ± standard deviation (SD).
